# Smell and taste function in childhood cancer patients: a feasibility study

**DOI:** 10.1007/s00520-020-05650-3

**Published:** 2020-08-02

**Authors:** Mirjam van den Brink, Irene IJpma, Britt van Belkom, Marta Fiocco, Remco C. Havermans, Wim J. E. Tissing

**Affiliations:** 1 Laboratory of Behavioural Gastronomy, Centre for Healthy Eating and Food Innovation, Maastricht University Campus Venlo, Venlo, Netherlands; 2 Princess Máxima Center for Pediatric Oncology, Utrecht, Netherlands; 3 Medical Statistics, Department of Biomedical Data Science, Leiden University Medical Center, Leiden, Netherlands; 4 Mathematical Institute, Leiden University, Leiden, Netherlands; 5 Department of Pediatric Oncology and Hematology, University of Groningen, Beatrix Children’s Hospital, University Medical Center Groningen, Groningen, Netherlands

**Keywords:** Smell, Taste, Childhood cancer, Chemotherapy

## Abstract

**Purpose:**

Chemotherapy can affect smell and taste function. This has never been investigated in childhood cancer patients during chemotherapy. The objective of this study was to determine whether psychophysical smell and taste tests are suitable for children with cancer. Taste and smell function, fungiform papillae density, and eating behavior were measured before (T1) and after (T2) a cycle of chemotherapy and compared with healthy controls.

**Methods:**

Thirty-one childhood cancer patients treated for a hematological, solid, or brain malignancy (median age 12 years, 16 girls), and 24 healthy controls (median age: 11 years, 10 girls) participated. Smell function was measured using Sniffin’ Sticks, including a threshold, discrimination, and identification test. Taste Strips were used to determine recognition thresholds for sweet, sour, salty, and bitter taste. Papillae density was investigated by counting the fungiform papillae of the anterior tongue. Eating behavior was assessed using the Behavioral Pediatrics Feeding Assessment Scale (BPFAS).

**Results:**

Smell and taste function could be investigated in more than 90% of the patients, while fungiform papillae density could be determined in 61% of the patients. A significant difference in smell threshold was found between patients and controls (*p* = 0.001), showing lower thresholds in patients. In patients, sweet taste (*p* < 0.001), bitter taste (*p* = 0.028), and total taste function (*p* = 0.004) were significantly different after a cycle of chemotherapy, with higher scores at T2.

**Conclusion:**

The assessment of smell, taste, and fungiform papillae density is feasible in children with cancer. Results of the current study suggest that smell and taste sensitivity increased in children with cancer.

## Introduction

Childhood cancer survival rates have markedly improved in recent decades [1]. Increased survival can be attributed to providing more intensive therapies. However, as a result, almost all such children suffer from bothersome or severe treatment-related side effects [2]. Nausea, vomiting, and loss of appetite are well-known side effects among childhood cancer patients, interfering with food intake [3]. Taste changes have been found to be the third most common bothersome symptom (prevalence 60.3%) [2]. These changes are an often overlooked side effect contributing to inadequate food intake, which in turn affects nutritional status [4]. Poor nutritional status in children with cancer is associated with increased infections, poor survival, and impaired health-related quality of life [5, 6].

Studies investigating changes in smell and taste among childhood cancer patients are rare. Skolin and colleagues found that children with cancer undergoing chemotherapy had significant lower scores for bitter taste and made more taste recognition errors compared with controls [7]. However, this cross-sectional study was heterogeneous regarding chemotherapy (i.e., patients receiving doxorubicin, methotrexate, ifosfamide, cytarabine, procarbazine, dacarbazine, cisplatin, or cyclophosphamide per protocol depending on diagnosis and treatment phase), and only ten patients (median age 14.5 years) underwent a taste test. Qualitative studies indicated that changes in taste were the predominant cause of eating problems and altered food preferences in children with cancer, although specific food choices were highly variable [7, 8]. Changes in taste are often accompanied by changes in smell function. This has been found in adult patients undergoing various chemotherapy regimens (e.g., anthracycline, taxane, platinum containing) but has not been studied in childhood cancer patients during chemotherapy [9]. Only one study evaluated both smell and taste function in pediatric patients (*n* = 10) after bone marrow transplantation, but not during chemotherapy [10]. As current evidence comes from small studies and lack the assessment of smell function in childhood cancer patients during chemotherapy, prospective studies are needed to measure smell and taste function in children with cancer during chemotherapy.

Before investigating smell and taste changes in childhood cancer patients extensively, it must be considered whether psychophysical smell and taste assessments can be obtained without unpleasant side effects. For example, if children with cancer are rather sensitive to odors, which are regularly seen in adult patients, they might experience nausea when certain odors are presented [11]. Therefore, this study aimed to examine whether measurements of smell, taste, and fungiform papillae density are feasible (i.e., completed by more than 60% of the patients) in children with cancer and if those tests require adjustments. Furthermore, smell and taste function, fungiform papillae density, and eating behavior were evaluated during chemotherapy (i.e., before and after a cycle) and compared with healthy controls, results of which contribute to a burgeoning understanding of smell and taste changes and their consequences in children with cancer.

## Methods

### Participants

This study was performed at the Princess Máxima Center for Pediatric Oncology in Utrecht, the Netherlands. Eligible patients were children diagnosed with a hematological, solid, or brain malignancy, currently treated with chemotherapy. Treatment regimens supplied during the study period can be found in Table 1.

**Table 1:** Characteristics of childhood cancer patients and healthy controls

Characteristics	Patients (*n* = 31)	Controls (*n* = 24)	*p* value
Gender, female (*n*, %)	16 (51.6)	10 (41.7)	0.464
Age (median, range)	12 (7–17)	11 (6–18)	0.658
6–8 years (*n*, %)	7 (22.6)	5 (20.8)	
9–14 years (*n*, %)	14 (45.2)	13 (54.2)	
15–18 years (*n*, %)	10 (32.2)	6 (25.0)	
Diagnosis			
Hematologic malignancy (*n*, %)	16 (51.6)		
ALL	8 (25.8)		
AML	1 (3.2)		
Lymphoma	7 (22.6)		
Brain tumor (*n*, %)	3 (9.7)		
Medulloblastoma	3 (9.7)		
Solid tumor (n, %)	12 (38.7)		
Bone	9 (29.0)		
Rhabdomyosarcoma	3 (9.7)		
Chemotherapy regimen (*n*, %)^*^			
Alkylating agents^a^	14 (53.8)		
Anthracyclines^b^	7 (26.9)		
Platinum agents^c^	4 (15.4)		
Vinca alkaloids^d^	15 (57.7)		
Antimetabolites^e^	11 (42.3)		
Epipodophyllotoxins^f^	5 (19.2)		
Other^g^	11 (42.3)		
Intensity of Treatment Rating (ITR)			
Moderate intensive (*n*, %)	11 (35.5)		
Very intensive (*n*, %)	17 (54.8)		
Most intensive (*n*, %)	3 (9.7)		

*ALL* acute lymphoblastic leukemia, *AML* acute myeloid leukemia

^*^Provided chemotherapy between T1 and T2, *n* = 26

^a^Cyclophosphamide, dacarbazine, ifosfamide, lomustine

^b^Doxorubicin

^c^Carboplatin, cisplatin

^d^Vincristine

^e^Methotrexate, 6-mercaptopurine

^f^Etoposide

^g^Asparaginase, dactinomycin, dexamethasone, prednisone

Patients were compared to healthy controls, matched by age and gender. Controls were recruited among siblings and friends of the patients. Participants were eligible for participation if they were between 6 and 18 years and able to understand Dutch. Exclusion criteria were as follows: isolated congenital anosmia (ICA) or a self-reported allergy to quinine.

### Procedure and feasibility assessment

Patients were measured twice whereas controls were measured only once. A measurement was postponed in the case of severe oral mucositis or having a cold. During the first measurement in patients (T1), which was performed at day one of a cycle of chemotherapy somewhere during treatment protocol, feasibility of the tests was assessed. A test was considered feasible if at least 60% of the patients could complete the test without unpleasant side effects. Additionally, patients were asked to rate the tests by using smileys regarding the following topics: fun, difficulty level, concentration, and time duration.

If the first measurement in patients was considered viable, a second measurement (T2) was performed to assess potential changes in smell and taste within a cycle of chemotherapy. When a patient was admitted to the hospital for at least 4 days, the second measurement was taken on the last day of admission. In case of a shorter hospital stay, the second measurement was performed on the first day of the following chemotherapy cycle (usually 21 days later).

### Measurements

#### Smell function

Sniffin’ Sticks (Burghart, Wedel, Germany) were used to determine smell function [12]. This test comprises three parts: odor threshold (THR), discrimination (DIS), and identification (ID). All odorants are presented in pen-like odor dispensing devices, which are positioned 2 cm in front of the patient’s nostrils for approximately 3 sec. For the THR-test, a modified set of eight dilutions of phenyl ethyl alcohol (PEA; rose-like smell) was used [13]. Each time, three pens, of which one contained PEA and two contained a non-odorous solvent, were presented to the blindfolded participant. The participant had to distinguish the odor-containing pen in a staircase up-down procedure by starting with the lowest concentration of PEA. Reversal of the staircase was triggered by two correct or one false identification until seven reversals were obtained or until five reversals if attentiveness waned. The average of the last four reversal points was used as threshold score and ranged between 1 and 15. For the DIS-test, 16 triplets, containing two equal odorants and one different odorant, were presented in a randomized order. Participants, who were blindfolded, had to determine which pen smelled differently. ID was assessed by presenting sixteen common odorants, and participants had to identify these odorants by using a four-choice task. For DIS and ID, a correct response resulted in one point and scores range between 0 and 16.

#### Taste function

Filter-paper strips (Taste Strips, Burghart, Wedel, Germany) with impregnated concentrations of sweet, sour, salty, and bitter were used to determine taste recognition thresholds [14]. Each time, one of four concentrations of sweet taste (0.05, 0.1, 0.2, and 0.4 g/ml sucrose), sour taste (0.05, 0.09, 0.165, and 0.3 g/ml citric acid), salty taste (0.016, 0.04, 0.1, and 0.25 g/ml sodium chloride), or bitter taste (0.0004, 0.0009, 0.0024, and 0.006 g/ml quinine hydrochloride) was presented in an order of increasing concentrations. Before the test began, the highest concentration of each taste was given to familiarize participants with the taste qualities. Taste strips were placed on the middle of the tongue for whole-mouth testing. Participants were then asked whether the perceived taste was sweet, sour, salty, bitter, or tasteless. Scores for each taste quality range from 0 to 4, and the total taste score was derived by summing the scores of each taste quality (range 0–16).

#### Subjective smell, taste, and appetite

Participants were asked to self-assess their smell, taste, and appetite on a 5-point Likert scale (1 “very bad” to 5 “very good”). In addition, participants rated their smell, taste, and appetite (1 “much worse” to 5 “much better”) compared with the start of chemotherapy (patients) or with the last month (controls).

#### Fungiform papillae density

Fungiform papillae density was investigated by staining the tongue with a 0.9% Brilliant Blue food dye (Pomona Aroma, Hedel, the Netherlands), diluted to a concentration of 1:10 at which fungiform papillae remain pink [15]. Participants were asked to extend their tongue and secure it gently between their teeth and lips. Subsequently, the tongue was dried with filter paper, stained with blue food dye, and dried again. Then, a 15-mm-diameter Whatman circular filter paper Grade 1 (GE Healthcare Life Science, Chalfont St. Giles, UK) with a 6-mm-diameter circular cut-out (area 0.283 cm^2^) was placed on the anterior of the left side of the tongue, next to the midline [16]. At least three close-up images of the tongue were taken by a digital camera (Canon Powershot SX70 HS, Tokyo, Japan). Afterwards, the clearest image was further investigated in Fiji, a distribution of ImageJ software (National Institutes of Health, Bethesda, USA) [17]. The Denver Papillae Protocol (DPP) was used for counting fungiform papillae [18].

#### Eating behavior

Eating behavior was assessed using the Behavioral Pediatrics Feeding Assessment Scale (BPFAS) [19]. The BPFAS is a 35-item parent-report questionnaire that consists of 25 items that focus on the child’s eating behavior and 10 items that focus on parents’ feeding strategies. For each statement, parents reported how often the particular behavior occurred on a 5-point Likert scale (1 “never” to 5 “always”). They were also asked to indicate whether they believed that this behavior was problematic or not. Four scores are thus generated: Child Behavior-Frequency (CBF) and Parent Behavior-Frequency (PBF) (which refer to how often the specific child and parent behavior occur) and Child Behavior-Problems (CBP) and Parent Behavior-Problems (PBP) (which reflect the number of behaviors seen as problematic). Higher scores indicate more eating/feeding problems [20].

#### Treatment intensity

Treatment intensity was rated with the Intensity of Treatment Rating scale (ITR-3), a psychometrically valid classification of pediatric cancer treatment, into one of four levels ranging from 1 “minimally invasive” (e.g., in case of stage 1 Wilm’s tumor) to 4 “most invasive” (e.g., in case of a brain tumor with treatment requiring HSCT) [21].

### Statistical analysis

Descriptive statistics are presented as median with interquartile range (IQR) or number of participants (N) with percentage (%) for both groups. The Mann-Whitney *U* Test was used to compare smell, taste, fungiform papillae density, and BPFAS scores between controls and patients at T1. The Wilcoxon signed-rank test was used to compare changes in smell, taste, and fungiform papillae density between the two measurements in patients. Spearman’s test was employed to investigate correlations between taste function and fungiform papillae density and taste function and eating behavior in patients at T1. A 5% alpha level was used. Data analysis was performed with IBM SPSS Statistics (version 25.0).

## Results

### Participant characteristics

Thirty-one patients and 24 healthy controls were included in this study (Table 1). After the first measurement, five patients left the study because they completed their treatment (*n* = 2), continued treatment somewhere else (*n* = 1), or became too ill (*n* = 2). Median time interval between T1 and T2 was 21 days (IQR 14–37). Six patients underwent the second measurement more than 37 days later due to postponed admissions or severe complications.

### Feasibility assessment in patients

Twenty-nine patients (94%) performed the THR-test, and for the 23 of them (79%), a THR-score could be obtained after seven reversals of the staircase. For the remaining six patients, the THR-score was calculated after five reversals of the staircase as their attentiveness waned. For DIS and ID, 28 (90%) and 30 (97%) patients could complete these tests, respectively. Thirty patients (97%) finished the taste test. One DIS-test and taste test were prematurely terminated due to nausea. For papillae density, six patients (19%) did not undergo the measurement. Reasons for not participating in this test were as follows: nausea/gagging (*n* = 2), anxiety/tension (*n* = 2), or logistical reasons (*n* = 2). From the remaining 25 patients, six photos were of insufficient quality to count the fungiform papillae. Overall, fungiform papillae density could be calculated for 19 (61%) of the patients.

Concerning patients’ experiences, 81% reported that they really liked the overall assessment and 84% reported that they did not experience any problems concerning concentration. Difficulty of the tests was qualified by 71% of the patients as “a bit difficult.” In addition, 39% of the patients reported time of the assessment as “long lasting.”

### Smell and taste function

**Fig. 1:**
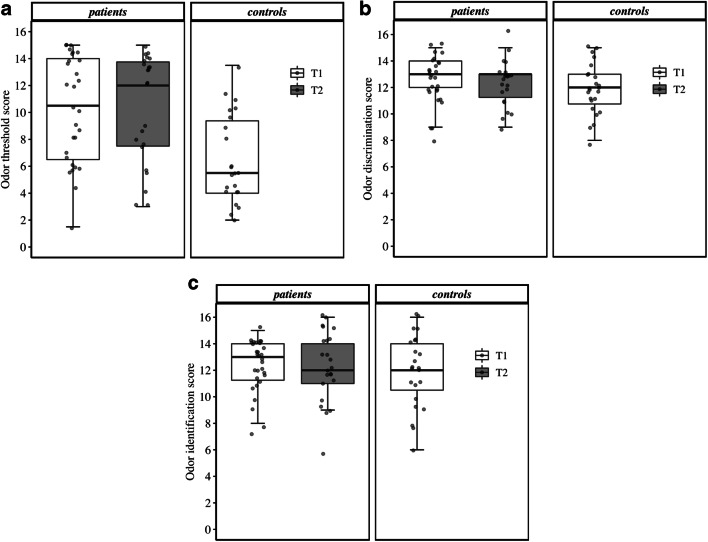
Boxplots for the three different smell tests: odor threshold (**a**), odor discrimination (**b**), and odor identification (**c**). The boxplots refer to the median score (midpoint of the scores), the first quartile of the scores (Q1, lower boundary of the box), and the third quartile of the scores (Q3, upper boundary of the box). The range of the box represents the interquartile range (IQR = Q3 – Q1), and the whiskers indicate what data points can be considered outliers. The upper whisker extends to the most extreme score no more than 1.5 times the IQR above Q3, and the lower whisker extends to the most extreme score no more than 1.5 times the IQR below Q1. Note that the data points represent individual scores and that these points were slightly jittered to avoid overplotting

Figure 1 shows smell function of the childhood cancer patients and controls. A significant difference in smell threshold was found between patients and controls (*p* = 0.001), showing lower thresholds in patients. DIS and ID were not significantly different between the two groups. In patients, no significant differences in smell function were found between the two measurements.

**Fig. 2:**
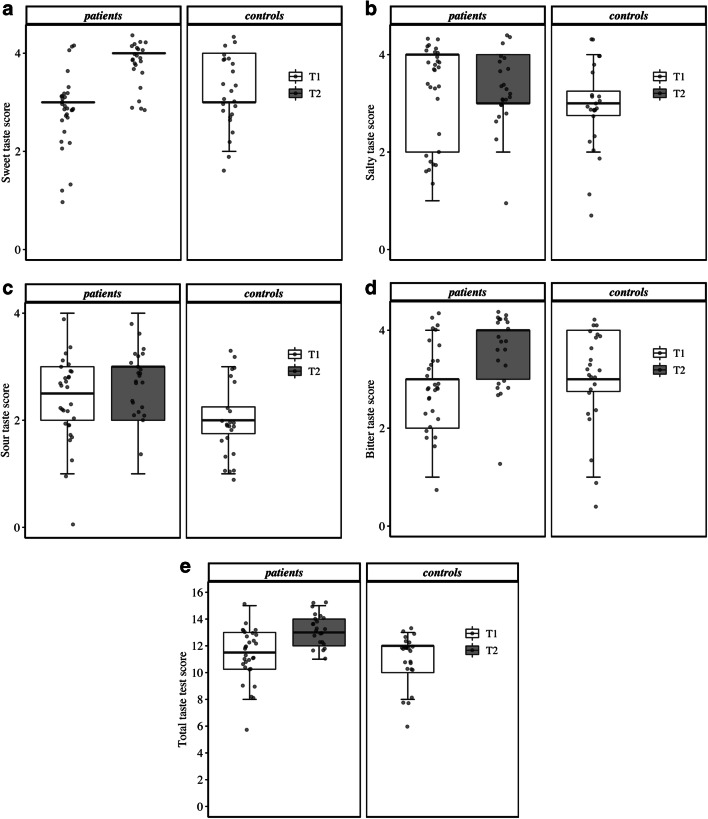
Boxplots for the “Taste Strips” test scores: sweet taste (**a**), salty taste (**b**), sour taste (**c**), bitter taste (**d**), and total score (**e**). Note that due to the limited range of possible scores for the individual taste qualities (0–4; **a–d**), some boxes (and whiskers) appear constricted

Compared with controls, patients had a different sour taste threshold (*p* = 0.042) (Fig. 2). Regarding the other taste qualities, no significant differences were found between patients and controls. In patients, sweet taste (*p* < 0.001), bitter taste (*p* = 0.028), and total taste function (*p* = 0.004) were significantly different after a cycle of chemotherapy, showing higher scores at T2.

Table 2 shows subjective smell, taste, and appetite of childhood cancer patients at T1. Twelve patients (39%) reported changes in smell, and 11 patients (36%) reported taste changes, reflecting both increased and decreased perceptions. In addition, 24 patients (77%) reported alterations in appetite.

**Table 2:** Subjective smell, taste, and appetite among childhood cancer patients at T1 (*n* = 31).

Rating	Number of patients (%)	Changes since start chemotherapy	Number of patients (%)
Smell			
Very good	8 (25.8)	Much better	2 (6.4)
Good	19 (61.3)	Better	6 (19.4)
Moderate	4 (12.9)	Unchanged	19 (61.3)
Bad	0 (0.0)	Worse	4 (12.9)
Very bad	0 (0.0)	Much worse	0 (0.0)
Taste			
Very good	6 (19.4)	Much better	1 (3.2)
Good	20 (64.5)	Better	4 (12.9)
Moderate	3 (9.7)	Unchanged	20 (64.5)
Bad	2 (6.4)	Worse	6 (19.4)
Very bad	0 (0.0)	Much worse	0 (0.0)
Appetite			
Very good	8 (25.8)	Much better	0 (0.0)
Good	12 (38.7)	Better	11 (35.5)
Moderate	6 (19.4)	Unchanged	7 (22.6)
Bad	3 (9.7)	Worse	10 (32.2)
Very bad	2 (6.4)	Much worse	3 (9.7)

### Fungiform papillae density

Fungiform papillae density was neither significantly different between patients and controls, nor between the two measurements in patients (Table 3). Fungiform papillae density was not significantly correlated with taste function in children with cancer.

**Table 3:** Median scores (IQR) of fungiform papillae in childhood cancer patients and healthy controls

	Controls (*n* = 17)	Patients (T1) (*n* = 19)	Patients (T2) (*n* = 18)	*p* value*
Number of papillae	15.0 (12.5–24.0)	20.0 (15.0–28.0)	20.5 (17.8–25.8)	0.327
Papillae density (cm²)	53.1 (44.2–84.9)	70.7 (53.1–99.0)	72.5 (62.8–91.1)	0.294

*IQR* interquartile range

*p value between T1 and T2 within patients

### Eating behavior

No significant differences were found in BPFAS scores and the prevalence of eating disorders between patients and controls (Table 4). In patients, the total taste function at T1 was negatively correlated with PBF (*r* = − 0.402, *p* = 0.042), meaning that a better taste function is associated with less frequently reported “poor” feeding strategies. Additionally, a difference in taste function (T1 vs T2; i.e., increased sensitivity in this case) was positively correlated with CBF (*r* = 0.469, *p* = 0.037). Thus, increased taste function in children with cancer was associated with eating disorders.

**Table 4:** Comparisons of BPFAS scores across childhood cancer patients and healthy controls

	Patients (*n* = 26)		Controls (*n* = 20)	
	Median (IQR)	*N* disorder (%)	Median (IQR)	*N* disorder (%)
Child Behavior-Frequency (CBF)	43.0 (38.5–47.3)	3 (11.5)	37.0 (34.3–45.8)	2 (10.0)
Child Behavior-Problem (CBP)	0.0 (0.0–0.8)^a^	3 (12.5)	0.0 (0.0–0.0)^b^	0 (0.0)
Parent Behavior-Frequency (PBF)	16.0 (13.0–18.0)	3 (11.5)	14.0 (12.0–16.0)	0 (0.0)
Parent Behavior-Problem (PBP)	0.0 (0.0–0.0)^a^	3 (12.5)	0.0 (0.0–0.0)^b^	1 (5.9)

*IQR* interquartile range

^a^*n* = 24

^b^*n* = 17

## Discussion

The present study has shown that assessing smell, taste, and fungiform papillae density is feasible in children with cancer, as more than 60% of the patients were able to complete the tests. Although feasible, some adaptions are deemed necessary regarding time duration and difficulty level of the tests. Furthermore, we showed that taste function increased in childhood cancer patients during chemotherapy, especially for sweet and bitter taste. Lower smell thresholds were found in patients compared with healthy controls, which suggest that both smell and taste sensitivity increased in children with cancer.

Regarding smell function, a wide step method was used for the threshold test to enhance concentration and reduce time of investigation. This method has never been used in children but has been shown reliable in adults [13]. Due to the size of our control group, and its distribution across different age categories, it was not possible to compare the threshold scores with those derived from a regularly used narrow step method [22]. Still, the wide step method provides an advantage for threshold testing in participants where time of investigation should be kept as short as possible [13]. Although only one discrimination test was prematurely terminated due to nausea, several patients noted that they did not like the intensity and large number of odorants either. Concerning odor identification, children were often not familiar with some of the odorants (e.g., turpentine, apple) from the odor identification test. This finding is consistent with a study among German children [23]. The Universal Sniff Test, a recently developed international odor identification test for children, will be more suitable as odorants are selected on familiarity [24]. This test is now commercially available, including normative values for children aged 6–17 years [22].

As smell thresholds are less influenced by age, contribute to a large extent to the diagnosis of smell loss, and seem affected the most in our study population, the assessment of smell function in children with cancer should include at least an odor threshold test [25, 26]. However, the assessment of several components of smell function, instead of a single component, is preferred. Therefore, a suitable odor identification task for children, such as the Universal Sniff Test, should be added. Odor discrimination does not seem to have much added value in children with cancer, and child-friendly tasks are lacking. Removing this task will save at least 10 min.

Investigating taste function and papillae density can be considered feasible, although the assessment of papillae density was more problematic in children with cancer. The main obstacle was not the measurement, which relatively few children disliked**,** but rather obtaining a proper photograph of the tongue. Photographs regularly failed due to movement of the tongue or being taken in poorly lit rooms. Sometimes, fungiform papillae were invisible because of a white layer on the surface of the tongue. The so-called oral thrush, or oral candidiasis, is common among people with a weakened immune system [27]. In addition, papillae density was not significantly different between the groups nor correlated with taste function in patients. Although feasible, the limitations and current results do not warrant further investigation of fungiform papillae density in children with cancer. Practical issues need to be overcome first to reduce the burden on children with cancer.

Results of our study seem to indicate that smell function sensitizes in children with cancer, showing lower smell thresholds compared with controls. Smell function did not change significantly after a cycle of chemotherapy in patients. Our findings are in contrast with those of previous studies who examined adults receiving chemotherapy. For example, women undergoing chemotherapy for breast cancer or gynecological malignancies showed increased smell thresholds during chemotherapy [9]. In addition, men undergoing chemotherapy for testicular cancer showed no changes in smell function [28]. Although there was no measurement before diagnosis, and it cannot be ruled out that lower smell thresholds were already present before diagnosis, several children with cancer (*n* = 8) reported a better or much better smell perception since the start of chemotherapy. This may well be an underestimation. Increased smell sensitivity was typically judged as negative. Possibly, some children conflated their evaluation of their altered sense of smell with their altered smell sensitivity leading them to rate their sense of smell as “worse” after chemotherapy. Future research on subjective smell and taste sensitivity in children with cancer requires more careful instruction and phrasing of questions.

The current study showed increased sweet, bitter, and total taste function after a cycle of chemotherapy. So far, evidence regarding smell and taste function in childhood cancer patients during chemotherapy is limited to cross-sectional studies with small sizes. Those studies generally show reduced taste perception for all taste qualities, or bitter taste only, in children with cancer compared with healthy controls [7, 29]. When reviewing prospective studies among adults receiving chemotherapy, changes in sweet taste and, to a lesser extent, bitter taste seem more common than changes in salt or sour perception [30]. However, taste changes in the current subset of childhood cancer patients were characterized by increased perception of sweet and bitter taste, while adults generally experience a decreased perception of these taste qualities during chemotherapy. Maybe other pathways are involved in children compared with adults.

The etiology of smell and taste changes during chemotherapy is not fully understood. In general, damage to sensory receptor cells and abnormal neuronal activity are thought to be the major cause of these distortions [31]. Smell and taste receptor cells have high turnover rates, as do cancer cells, and particularly rapidly dividing cells are affected by chemotherapy. With respect to specific chemotherapeutic substances, drugs such as methotrexate, vincristine, cisplatin, carboplatin, doxorubicin, cyclophosphamide, 6-mercaptopurine, and 5- fluorouracil all seem to be associated with taste changes in adults but not necessarily with smell changes [32]. Taste changes may be also related to oral mucositis, poor oral hygiene, infections, or a dry mouth. In addition, it is presumed that cancer-related inflammation can trigger apoptosis of the taste bud cells through cytokine signaling pathways, thereby contributing to the development of taste disorders [33]. An enhanced ability to smell during chemotherapy, potentially resulting in food aversions and nausea, might be a strengthened defense mechanism of the sensory organ to avoid ingestion of potentially harmful substances into the body [34]. However, many questions remain regarding smell and taste changes during chemotherapy.

Taste function was correlated with eating behavior and feeding strategies in children with cancer. This is in line with qualitative studies that already highlighted the influence of taste changes on food preferences and eating behavior [7, 8, 35]. Since eating behavior and food preferences are still developing in children, and are strongly influenced by the chemical senses, it is suggested that the impact of smell and taste changes in the long term could be large as well [36, 37]. To prevent children with cancer from inadequate food intake and bad dietary habits due to this phenomenon, longitudinal studies are needed to identify the course of smell and taste changes and its consequences regarding food intake and eating behavior during and after chemotherapy.

This study aimed to investigate feasibility of smell, taste, and papillae density assessment in children with cancer. Therefore, the current results regarding smell and taste function do not allow for strong conclusions and should be considered tentative. Even if it is the largest study to date, the size of the current study is small, lacks a measurement at diagnosis, and varies in time intervals between measurements. Nevertheless, the prospective study design and control group make the results of this feasibility study already useful for a burgeoning understanding of smell and taste changes in children with cancer during chemotherapy.

In conclusion, the assessment of smell and taste function and fungiform papillae density is feasible in children with cancer. Future longitudinal studies should focus on smell (threshold and identification) and taste function in children with cancer, whereas the assessment of fungiform papillae density should be omitted. In addition, results of the current study suggest a remarkable increased smell and taste sensitivity in children with cancer, which was an unexpected finding and requires further investigation.

## Data Availability

Data that support the findings of this study are available from the corresponding author upon reasonable request.
